# Deinoxanthin Recovers H_2_O_2_-Stimulated Oxidative Complications of Bone Marrow-Derived Cells and Protects Mice from Irradiation-Mediated Impairments

**DOI:** 10.3390/antiox14101180

**Published:** 2025-09-26

**Authors:** Govinda Bhattarai, Sung-Ho Kook, Saroj Kumar Shrestha, Jeong-Hwan Park, Shankar Rijal, Gyeongho Tae, Doyoung Hwang, Seung-Moon Park, Jeong-Chae Lee, Young-Mi Jeon

**Affiliations:** 1 Cluster for Craniofacial Development and Regeneration Research, Institute of Oral Bioscience, School of Dentistry, Jeonbuk National University, Jeonju 54896, Republic of Korea; govinda@jbnu.ac.kr (G.B.); kooksh@jbnu.ac.kr (S.-H.K.); choosey95@naver.com (J.-H.P.); 2 Research Center of Bioactive Materials, Department of Bioactive Material Sciences, Jeonbuk National University, Jeonju 54896, Republic of Korea; rijal49@jbnu.ac.kr; 3 Department of Biochemistry and Molecular Genetics, University of Alabama at Birmingham, Birmingham, AL 35233, USA; skshrest@uab.edu; 4 Department of Bioenvironmental Chemistry, Jeonbuk National University, Jeonju 54896, Republic of Korea; rudgh2360@jbnu.ac.kr (G.T.); hdy0928@jbnu.ac.kr (D.H.); smpark@jbnu.ac.kr (S.-M.P.); 5 Research Institute of Clinical Medicine of Jeonbuk National University, Biomedical Research Institute of Jeonbuk National University Hospital, Jeonju 54907, Republic of Korea

**Keywords:** deinoxanthin, hydrogen peroxide, total body irradiation, bone marrow-derived cells, antioxidant potency, radioprotection

## Abstract

A growing interest is focused on the efficient production of deinoxanthin (DEIX) and its use as a bioactive antioxidant. Here, we produced DEIX from *Deinococcus radiodurans* and examined how DEIX regulates hydrogen peroxide (H_2_O_2_)-mediated oxidative behaviors in mouse-derived bone marrow (BM) stromal cells and BM monocytes. We also evaluated whether oral supplementation with DEIX has radioprotective potential against total body irradiation (TBI)-mediated impairments in growth, organs, survival, and hematopoietic development using a mouse model. The direct addition of DEIX recovered H_2_O_2_-mediated oxidative disorders in the proliferation and the balance between osteoblast and osteoclast activity of the BM-derived cells in a dose-dependent manner. We found that recovery was closely associated with the DEIX’s potencies to remove cellular reactive oxygen species and to restore the expression of key molecules that tightly control bone homeostasis. Long-term oral supplementation with DEIX (25 mg/kg body weight, once per day for 42 consecutive days) protected mice against sub-lethal TBI (5 Gy)-mediated decreases in organ and body weights and lifespan. Supplemental DEIX also inhibited TBI-mediated structural damage in organs and restored endogenous antioxidant defense systems in the liver of TBI-exposed mice. Moreover, supplemental DEIX recovered a dysregulated hematopoietic process in TBI-exposed mice. Collectively, our results introduce an efficient method to produce DEIX and demonstrate its potency to recover oxidative cellular complication in H_2_O_2_-exposed BM-derived cells. Overall, our findings suggest that DEIX is a great antioxidative molecule to prevent or protect TBI-mediated systemic damages.

## 1. Introduction

Approximately 90% of endogenously generated-reactive oxygen species (ROS) are produced from the mitochondrial electron transfer chain [1]. ROS at physiologic levels modulate cell fate and behavior and mediate cellular communication and immunomodulation [2]. Various biological processes, including proliferation, differentiation, and survival of cells are also affected in relation to the levels of intracellular ROS [3]. The levels of ROS are tightly controlled by the primary antioxidant systems consisting of enzymatic antioxidants, such as superoxide dismutases (SODs), catalase (CAT), and glutathione peroxidase (GPx), as well as non-enzymatic ROS scavengers, such as reduced glutathione, ascorbic acid, α-tocopherol, and carotenoids [4,5]. However, an excessive generation of ROS or a deficiency in antioxidant defense system can cause oxidative stress within cells. A prolonged and persistent oxidative stress destructs the structure of cellular macromolecules, such as lipids, proteins, and nucleic acids and eventually leads to cell death [6,7]. Oxidative stress is also implicated in the progression of various diseases, including Alzheimer’s disease, cancer, heart disease, and neurodegenerative disorders [8,9,10].

On the other hand, total body irradiation (TBI)-derived radiotherapy, a common treatment for cancer patients and those requiring bone marrow (BM) transplantation, has adverse effects, such as internal organ injury, abnormal hematopoietic development, and stem cell dysfunction [11,12,13]. TBI-mediated impairments are triggered by the increases in ROS and ROS-associated inflammatory mediators [14,15,16]. It is considered that BM-conserved stem cells such as hematopoietic stem cells (HSCs) and mesenchymal stem cells are sensitive to oxidative stress [14,17]. TBI-induced oxidative damage and fibrosis in organs are also a major limitation of radiotherapy [18].

Because oxidative stress is important challenge in maintaining cellular homeostasis, as well as in TBI-derived radiotherapy, studies have tried to develop various bioactive substances that can act as secondary antioxidants capable of directly removing ROS or activating the enzymatic antioxidant defense system. Many studies have demonstrated that naturally occurring phenolic compounds have good bioactivities in ameliorating or protecting cells against oxidative damage [19,20,21]. The bioactivity of phenolic compounds is closely associated with their abilities to scavenge intracellular ROS [22], maintain the stemness of cells [23], and activate anti-aging and anti-inflammatory signaling pathways [24]. In addition to phenolic compounds, carotenoids are potent antioxidants that exhibit biological, medicinal, and pharmacological activities [25]. Among the carotenoid compounds, deinoxanthin (DEIX; (2R)-2,1′-dihydroxy-3′,4′-didehydro-1′,2′-dihydro-β,ψ-caroten-4-one) is a unique xanthophyll carotenoid synthesized by *Deinococcus* microorganisms and exerts a radioprotective ability against even a high-dose irradiation [26]. DEIX was first characterized by *Deinococcus radiodurans* (*D. radiodurans*) and showed stronger antioxidant potential in scavenging singlet oxygen and hydrogen peroxide (H_2_O_2_) than other xanthophyll carotenoids such as carotene, lutein, lycopene, and zeaxanthin [27]. Its great antioxidant potential is associated with a unique structural feature [26]. Taken as a whole, it is suggested that DEIX prevents or attenuates oxidative cellular injury and TBI-mediated degenerative impairments.

As a growing interest is focused on a clinical use of DEIX, studies have tried to optimize culture conditions [28] or use systematic metabolic engineering to produce large amounts of DEIX [29]. Here, we efficiently produced DEIX by modifying the methods described previously [28] and examined its direct effects on H_2_O_2_-mediated functional loss of BM stromal cells (BMSCs) and BM monocytes (BMMs). We also evaluated whether oral supplementation with DEIX has radioprotective potential against TBI-mediated impairments in growth, organs, survival, and hematopoietic development. Consequently, we demonstrate that the addition of DEIX directly recovers H_2_O_2_-mediated oxidative disorders in the proliferation and the balance between osteoblast and osteoclast activity of BM-derived cells. We also highlight the protective potential of DEIX on TBI-mediated organ damage and lethality in a mouse model. Overall, our findings provide an efficient method to produce and isolate DEIX and suggest its usefulness as a therapeutic antioxidant to ameliorate or prevent oxidative stress-associated complications.

## 2. Material and Methods

### 2.1. Chemicals and Laboratory Equipment

Antibodies specific to nuclear factor erythroid 2-related factor 2 (Nrf2; BS1258) and runt-related transcription factor 2 (RUNX2; BS2831) were purchased from Bioworld Technology, Inc. (St. Louis Park, MN, USA). The receptor activator of the nuclear factor (NF)-κB ligand (RANKL; ALX-804-243) was purchased from Enzo Life Sciences, Inc. (Farmingdale, NY, USA). Osteopontin (OPN; ab8448) and heme oxygenase-1 (HO-1; ab13248) antibodies and 2′,7′-dichlorodihydrofluorescein-diacetate (DCF-DA) were obtained from Abcam (Cambridge, UK). Antibodies specific to cathepsin K (sc-4835), β-actin (sc-47778), c-Fos (sc-166940), and matrix metalloproteinase-9 (MMP-9; sc-393859) were acquired from Santa Cruz Biotechnology (Santa Cruz, CA, USA). Fetal bovine serum (FBS) was purchased from HyClone Laboratories (Logan, UT, USA) and antibiotic–antimycotic (2441713) was purchased from Gibco (Life Technologies, Carlsbad, CA, USA). Unless specified otherwise, other chemicals, antibodies, and laboratory consumables were purchased from Sigma-Aldrich Co. LLC (St. Louis, MI, USA), Abcam, and Falcon Labware (BD Biosciences, Franklin Lakes, NJ, USA), respectively.

### 2.2. Source of D. radiodurans and Its Flask and Bioreactor Fermentation

*D. radiodurans* was obtained from the area of Jeonju (Republic of Korea). In brief, ten soil samples (3 g/sample) were collected around the city and aliquoted into 15 mL tubes. To selectively isolate radiation-resistant *D. radiodurans*, each sample (1 g/sample) was exposed to 3 kGy/h γ-rays for 15 h using a cobalt-60 γ-irradiator (Model IR-221, MDS Nordion, Ottawa, Canada). In this study, the *D. radiodurans* was cultivated in TGY medium (0.5% tryptone, 0.1% glucose, and 0.3% yeast extract), and a single colony was obtained from TGY medium containing 1.5% agar after 48 h of incubation at 30 °C. A fresh single colony of *D. radiodurans* was inoculated into 35 mL of TGY medium in a 300 mL baffled flask and cultivated at 30 °C/250 rpm until the optical density of the seed culture reached 1.0–1.2 at 600 nm. Bioreactor batch fermentation was performed by transferring the seed cultures (1%) to a bioreactor (5L Biocanvas, Centrion, Incheon, Republic of Korea) containing 3 L of modified TY medium supplemented with 5 g/L tryptone, 5 g/L yeast extract, 10 g/L sucrose, 0.5 g/L MgSO_4_, and 1 mg/L MnCl_2_. Cultures were maintained at pH 7.4 for 48 h at the conditions of 30 °C, 300–350 rpm, and more than 30% of dissolved oxygen.

### 2.3. Extraction, Isolation, and Purification of DEIX

Cultured cell pellets were collected by centrifugation at 7000 rpm/4 °C for 20 min, washed twice with sterile water, and stored at −20 °C before the process for extraction. To extract carotenoids, the cell pellets were mixed with ice-cold acetone/methanol (7:2, *v*/*v*), homogenized for 10 min, and centrifuged at 4000 rpm for 20 min in a dark room. The colored supernatants were harvested, and the remaining pellets received the same processes 3–4 times until the red color mostly disappeared from the pellets. The collected supernatants were concentrated using a rotary evaporator (N1300, EYELA, Tokyo, Japan) in the conditions of 40 °C, 60–100 rpm, and 130–190 hpa for 25–30 min. The concentrated extracts were mixed with an equal volume of 5 N NaCl and 10% (*v*/*v*) ethyl acetate and stirred vigorously. The upper colored phase was collected, mixed with MgSO_4_, and evaporated using the evaporator in the same conditions (40 °C, 60–100 rpm, and 130–190 hpa) to yield the crude carotenoid extract. That crude extract (1.77 g) was dissolved in *n*-hexane:acetone (2:1, *v*/*v*) and underwent silica gel adsorption column chromatography (3.0 × 45 cm, 63–200 mm mesh, Merck, Darmstadt, Germany) and then elution with 300 mL of the organic solvent mixture [*n*-hexane:acetone (2:1, *v*/*v*)]. The color of each fraction (5 mL) was analyzed using thin-layer chromatography (TLC), and the fractions containing DEIX were collected, evaporated, and stored at −80 °C until use.

### 2.4. Identification of DEIX

The identification of the purified DEIX sample was performed using high-performance liquid chromatography (HPLC, UHPLC 3000, Thermo Fisher Scientific, Waltham, MA, USA) equipped with a Zorbax Eclipse XDB-C18 column (4.6 × 150 mm, 0.5 μm; Agilent Corp., Santa Clara, CA, USA) and a UV/VIS detector at 480 nm. An organic solvent mixture containing acetonitrile, methanol, and isopropanol (40:50:10, *v*/*v*/*v*) was used as the mobile phase, with a flow rate of 1 mL/min, injection volume of 10 μL, and column temperature at 40 °C. A Liquid Chromatography Mass Spectrometry (LC-MS) analysis was also carried out using a Hybrid Tandem LC/MS/MS spectrometer (Synapt G2-Si HDMS, Waters, Milford, MA, USA) equipped with an ACQUITY UPLC HSST3 column (100 mm × 2.1 mm, 1.8 μm, Waters) to determine the molecular weight of DEIX in the purified sample. As the mobile phase, a mixed solution of water and acetonitrile containing formic acid was added to the total solution to 0.1%, and the flow rate was 0.2 mL/min, injection volume was 10 μL, and column temperature was 40 °C. Ionization was analyzed in positive ion mode using electrospray ionization operated under a capillary voltage of 2.5 kV, MS scan range of 500–600 Da, source temperature of 120 °C, and prove temperature of 400 °C. The collision gas used was Ar.

### 2.5. Isolation and Culture of BM-Derived Cells

Whole BM cells were isolated from the femurs and tibias of young B6 mice (four week-old). The cells were resuspended in alpha-minimum essential medium (αMEM, Thermo Fisher Scientific) and centrifuged at 2000× *g* for 3 min. The pellets were spread onto 60 mm culture plates and incubated in growth medium (αMEM supplemented with 2 mM glutamine, 100 IU/mL penicillin G, 100 μg/mL streptomycin, and 20% FBS). On the second day, the non-adherent supernatant cells were collected to use as BMMs, and the adherent cells received additional incubation to use as BMSCs.

### 2.6. Proliferation Assay

BMSCs were seeded onto 96-well plates (2 × 10^3^ cells/well) in growth medium containing 1% FBS. Cells were incubated for 12 h and then DEIX was added at different concentrations (0–30 μM). After an additional incubation of 24 or 48 h, proliferation rate of the cells was assessed using a Cell Counting Kit-8 (CCK-8; Dojindo Lab, Rockville, MD, USA) according to the manufacturer’s instructions.

### 2.7. Flow Cytometric Assay

To assay the ability of DEIX to diminish intracellular ROS accumulation, BMSCs were seeded onto 6-well culture plates and incubated in a growth medium for 24 h. The culture medium was freshly replaced with growth medium containing 1% FBS and supplemented with 250 μM H_2_O_2_, DEIX (0–20 μM), or both. After an additional 24 h incubation, the cultures were treated with 10 μM DCF-DA for 30 min, and DCF-specific green fluorescence intensity at 515 nm (FL-1H) was recorded from 10,000 events/sample by flow cytometry (BD Aria III).

### 2.8. Western Blot Assay

To evaluate the effect of DEIX on the induction of molecules specific to antioxidation and osteogenesis, 6-well-seeded BMSCs were incubated in the presence and absence of 250 μM H_2_O_2_, DEIX (0–30 μM), or both. After 48 h of incubation, protein lysates were isolated from the cells, and the levels of HO-1, Nrf2, OPN, and RUNX2 were determined by Western blotting. In addition, the direct effects of DEIX on osteoclastogenic marker expression were determined using young B6 mice-derived BMMs. In this assay, BMMs were seeded onto 6-well culture plates (2 × 10^6^ cells/well) in the presence of 50 ng/mL RANKL, 250 μM H_2_O_2_, and/or DEIX (0–24 μM). After two days of incubation, the BMMs were processed for protein lysate extraction and immunoblotting, in which the levels of MMP-9, c-Fos, RANKL, and cathepsin K were evaluated after normalizing their band intensities to that of β-actin. For Western blot assay, BMSCs or BMMs were lysed in a cocktail buffer containing protease/phosphatase inhibitor (Cell Signaling Technology, Danvers, MA, USA). The protein extracts (15–20 µg/sample) were separated through sodium dodecyl sulfate–polyacrylamide gel electrophoresis on 10–12% gels and electroblotted onto polyvinylidene difluoride membranes. The blots were washed with a buffer including 10 mM Tris-HCl (pH 7.6), 150 mM NaCl, and 0.05% Tween-20 and blocked in 5% skim milk for 1 h before incubation with primary antibodies at the dilutions of 1:500 or 1:2500. The immunoreactive bands on the membranes were visualized using an enhanced peroxidase detection kit (ELPIS-Biotech, Daejeon, Republic Korea) and then exposed to a chemiluminescence imaging system (Vilber Lourmat, Collegien, France).

### 2.9. Mineralization Assay

To evaluate the direct effect of DEIX on the mineralization of BMSCs under oxidative stress, BMSCs were incubated in osteogenic medium supplemented with DAG (100 nM **d**examethasone, 50 µM **a**scorbic acid, and 10 mM β-**g**lycerophosphate) in combination with 250 μM H_2_O_2_, DEIX (0–20 μM), or both. The culture medium was replaced every three days during the incubation. After 21 days of incubation, the degree of mineralization was determined by staining the cells with alizarin red S (ARS) after 20 min of fixation in 70% ethanol. The red dye-stained cells were photographed using a light microscope (EL-Einsatz 451888, Carl Zeiss, Ostalbkreis, Germany). The ARS-stained cells were also treated with 10% acetylpyridinium chloride, and the amount of red dye was quantified by measuring the dye-specific optical density at 405 nm using a microplate reader (SPECTROstar^®^ Nano, Ortenberg, Germany).

### 2.10. Osteoclast Differentiation Assay

BMMs were seeded onto 6- (2 × 10^6^ cells/well) or 12-well culture plates (1 × 10^4^ cells/well) in the presence of 50 ng/mL RANKL, 250 μM H_2_O_2_, and/or DEIX (0–20 μM). After six days of incubation, the cultures were fixed with 4% paraformaldehyde in phosphate-buffered saline (PBS) and stained with a TRAP staining kit (LOT# UIQ-04, Cosmo Bio Co., Tokyo, Japan) according to the manufacturer’s instructions. The TRAP-stained cells were photographed using a light microscope, and multinucleated cells with more than three nuclei were counted as osteoclasts. The number and mean diameter of the osteoclasts were determined from the optic images.

### 2.11. RNA Isolation and qRT-PCR Assay

Total RNAs were extracted from BMSCs using Trizol reagent (Invitrogen, Waltham, MA, USA). RNA samples (1 μg/sample) were applied for cDNA synthesis using AmpiGene cDNA synthesis Kit (Enzo Life Sciences) according to the manufacturer’s instruction. qRT-PCR was performed with Power SYBR Green PCR Master Mix (Applied Biosystems, Waltham, MA, USA) and ABI StepOnePlus Real-Time PCR System (Applied Biosystems). The thermocycling conditions followed the pre-denaturation at 95 °C for 10 min and the amplification using three-step cycles of denaturation at 95 °C for 15 s, annealing at 60 °C for 30 s, and extension at 72 °C for 30 s for 40 cycles. Oligonucleotide primers used in this study were designed as 5′-cac atc cag tca gaa acc agt gg-3′ and 5′-gga atg tct gcg cca aaa gct g-3′ (NM_006164) for *Nrf2*, 5′-cca ggc aga gaa tgc tga gtt c-3′ and 5′-aag act ggg ctc tcc ttg ttg c-3′ (NM_002133) for *HO-1*, 5′-gag gga cta tgg cgt caa aca-3′ and 5′-gga tcc caa aag aag ctt tgc-3′ (XM_006523548.2) for *RUNX2,* 5′-acg gac agc tgg cac acc ag-3′ and 5′-ctc aca cac tcg gtt gtg gg-3′ (NM_008764.3) for *OPN*, and 5′-tcc aac gag atc gag atc c-3′ and 5′-aag ccg aat tcc tgg tct-3′ (NM_000088.4) for type I collagen (*COL1A1*). The level of glyceraldehyde 3-phosphate dehydrogenase (GAPDH; NM_002046) was considered as the endogenous reference during the quantification.

### 2.12. Animal and Ethics Statement

The male C57BL/6 mice (three or six weeks old) were purchased from Damul Science (Daejeon, Republic of Korea) and acclimatized for seven days before use. During the experimental periods, all animals were housed at 22 ± 1 °C, 55% ± 5% humidity, and a 12 h light/dark autocycle with ad libitum feeding in the Animal Center of the School of Dentistry, Jeonbuk National University. This study was carried out in strict accordance with the recommendations in the Animal Care and Use Guide of Jeonbuk National University. All experimental procedures were approved by the University Committee on Ethics in the Care and Use of Laboratory Animals (Approval No.: NON2024-043-003, Approval date: 16 April 2024).

### 2.13. DEIX Administration, TBI, and Sample Preparation

To evaluate the effects of supplemental DEIX on the weights of body and organs, the structure or function of organs, and the levels of circulating blood cells, mice (7 week-old) were divided into four groups (10 mice/group): non-TBI mice (control group), TBI-exposed mice who received oral gavage of 100 μL of olive oil (vehicle) (TBI group), TBI-exposed mice who were orally treated with DEIX (TBI + DEIX group), and non-TBI mice administered with DEIX alone (DEIX group). The TBI + DEIX and DEIX group received DEIX (25 mg/kg body weight) once per day for 42 consecutive days, from 7 days before to 35 days after TBI, and the TBI group received only the vehicle on the same days. The TBI and TBI + DEIX groups were exposed to 5 Gy TBI with γ-rays by regulating the dosage time (0.66 Gy/min) using the radioactive half-life of γ-rays on a rotating platform (Model IR-221, MDS Nordion). The oral gavage of olive oil (100 μL) itself did not cause any toxic effects to mice [30]. The amount of supplemental DEIX was adjusted as the body weights of the mice changed. Body weight was checked every week. Organs, including liver, kidney, spleen, and thymus were collected 12 h after the final administration of DEIX and used for further analyses. Peripheral blood was also collected from the mice one and five weeks after TBI via a tail vein cutting and used to count circulating blood cells.

### 2.14. Survival Study

The male C57BL/6 (7 week-old) were divided into the four groups (10 mice/group) and administered to vehicle or DEIX before and after TBI for the same days as described above. The survival rate was monitored in all groups every day after TBI up to 16 months post-TBI. The result was represented as survival rate (%) of total mice (*n* = 10)/group per month after TBI.

### 2.15. Histological Analyses

Organs such as liver, kidney, spleen, and thymus were isolated from the mouse groups and fixed with a 4% paraformaldehyde solution for 48 h. The tissues were dehydrated in an alcohol series, embedded in paraffin, and sectioned into 5.0 µm thicknesses. For hematoxylin and eosin (H&E) staining, tissue sections were treated with hematoxylin (Gill No. 3) before being counterstained with 0.25% Eosin Y stain (Thermo Fisher Scientific). A portion of each kidney tissue sample was stained with Masson’s trichrome (MT). The expression patterns of HO-1, SOD-1, and tumor necrosis factor α (TNF-α) in the liver tissue samples were evaluated by immunohistochemistry (IHC). In the IHC assay, tissue sections were stained with each primary antibody (1:200–500 dilutions) specific to the molecules, and their expression patterns were determined using rabbit-anti- or mouse-anti-Vectastain ABC DAB-HRP kits (CAS:SK-4100, Vector Laboratories, Burlingame, CA, USA). All procedures for IHC staining followed the relevant manufacturer’s instructions, and the stained sections were observed and photographed under a Motic slide scanner (Motic Sci & Tech Co., Ltd., Scottsdale, AZ, USA).

### 2.16. Assays for Enzyme Activities in Liver Tissue

Liver tissues were perfused with PBS and homogenized in 50 mM KH_2_PO_4_ solution for 5 min using a homogenizer (PRO Scientific Inc., Oxford, CT, USA) or in 200 μL of cold assay buffer provided by BioAssay Systems (Hayward, CA, USA). The homogenates were centrifuged at 14,000× *g* for 10 min, and the supernatants were collected to determine the activity of SOD, CAT, and GPx. SOD activity was analyzed using an assay kit (No. 706002, Cayman Chemical, Ann Arbor, MI, USA), and CAT and GPx activity was measured using an EnzyChrom™ CAT assay kit (ECAT-100; BioAssay Systems) and GPx assay kit (EGPX-100; BioAssay Systems), respectively, according to the manufacturer’s instructions.

### 2.17. Counting of Blood Cells

Peripheral blood was collected from the mice 7 and 35 days after TBI via a tail vein cutting and placed in K_2_EDTA-treated tubes (BD Biosciences). The levels of circulating white blood cells (WBCs), granulocytes, lymphocytes, and platelets were analyzed using an automated blood cell counter (PE-6800VET, Shenzhen Prokan Electronics Inc., Shenzhen, China).

### 2.18. Micro-Computed Tomography (μCT) Analysis

The femurs from the mouse groups were scanned using a desktop scanner (1076 Skyscan Micro-CT; Bruker, Kontich, Belgium) and analyzed with CTAn software (ver. 1.18) as described previously [30]. Here, the X-ray source was set at 75 KV and 100 μA with a pixel size of 18 μm. The image slices were reconstructed using a reconstruction software (NRecon software ver. 1.6.10.5). The values of bone parameters such as bone volume (BV, mm^3^) and bone volume percentage (BV/TV, %) were calculated.

### 2.19. Statistical Analyses

All data are represented as the mean ± standard deviation. Differences between two groups were analyzed by unpaired Student’s *t*-test using the GraphPad Prism (ver. 9.5) program (Boston, MA, USA). One-way analysis of variance (ANOVA) followed by Tukey’s multiple comparisons test was used in the same program to compare more than two groups. A value of *p* < 0.05 was considered statistically significant.

## 3. Results

### 3.1. Biosynthesis and Characterization of DEIX

**Figure 1:**
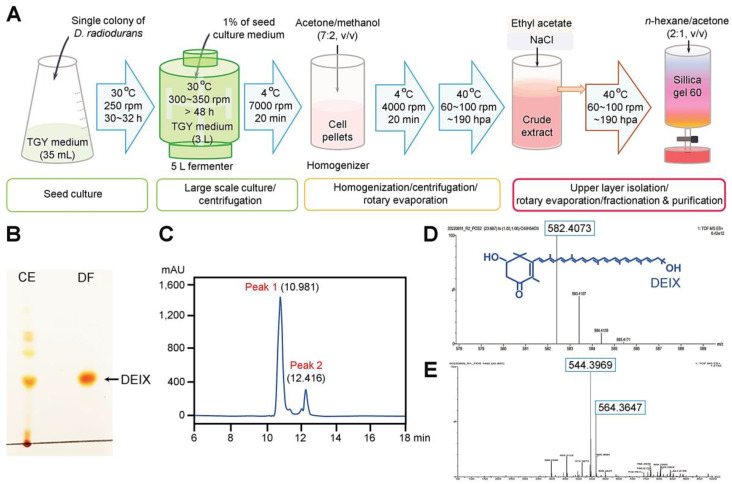
Production and characterization of DEIX. (**A**) A schematic diagram illustrating the processes for culturing and homogenizing *D. radiodurans* and purifying DEIX from the culture extracts. Identification of DEIX by (**B**) TLC, (**C**) HPLC, and (**D**,**E**) LC/MS/MS analyses, along with its chemical structure. CE; crude extract, DF; DEIX-containing fraction.

Figure 1A shows a schematic diagram representing the processes for the seed and large-scale cultures of *D. radiodurans*, homogenization, and the silica gel isolation and purification of DEIX. After silica gel column chromatography, DEIX was identified by comparing the number of compounds and the purity of each compound in the crude extract with the DEIX-containing fraction via TLC (Figure 1B). As shown in the TLC image, the crude extract displayed several colored compounds, whereas the DEIX-containing fraction showed only a DEIX-corresponding colored spot. The HPLC analysis of the crude extract exhibited two main polar peaks (Figure 1C). The original data of LC/MS/MS analysis on peak 1 and 2 are provided in Figure 1D,E. The carotenoid corresponding to peak 1 was identified as DEIX with the main fragmentation pattern of *m*/*z* 582.4, whereas peak 2 was proposed to contain DEIX derivatives such as phytoene (*m*/*z* 544.4) and 2-deoxydeinoxanthin (*m*/*z* 564.3).

### 3.2. The Direct Addition of DEIX Increases Proliferation of BMSCs and Inhibits H_2_O_2_-Mediated Oxidative Stress in the Cells

**Figure 2:**
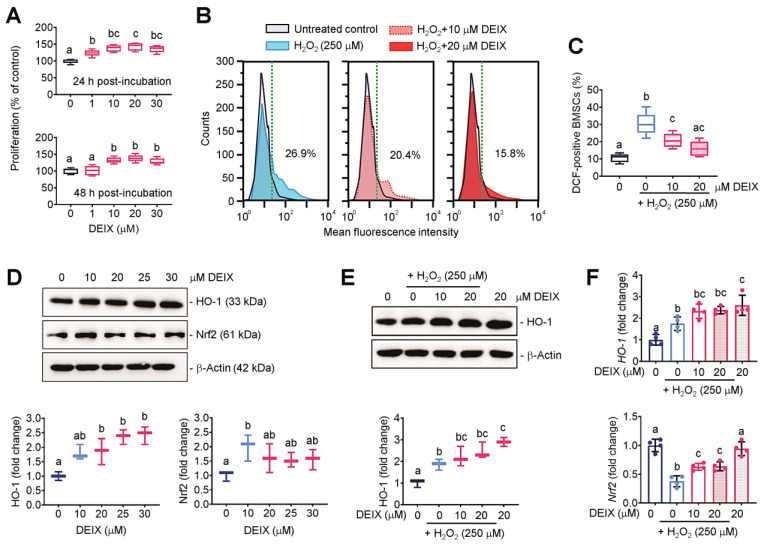
The addition of DEIX increases proliferation of BMSCs, but suppresses ROS accumulation and recovers the Nrf2/HO-1 pathway in H_2_O_2_-exposed BMSCs. BMSCs were isolated from young B6 mice that did not receive any treatment and then used to evaluate the direct effect of DEIX on H_2_O_2_-mediated cellular responses. (**A**) CCK-8 assay results showing the proliferation rate of BMSCs in relation to the concentrations of DEIX at 24 and 48 h of incubation (*n* = 5). (**B**) Flow cytometric histograms exhibiting the H_2_O_2_-mediated increase in DCF-positive BMSCs and its inhibition by DEIX treatment along with (**C**) the mean percentage of DCF-positive cells in relation to the treatment (*n* = 5). (**D**) Western blot images showing the expression levels of HO-1 and Nrf2 in BMSCs in relation to the concentrations of DEIX together with the graphs derived from densitometric analysis (*n* = 3). (**E**) Western blot result showing the level of HO-1 in BMSCs exposed to H_2_O_2_, DEIX, or both (*n* = 3). (**F**) qRT-PCR results exhibiting the expression of *HO-1* and *Nrf2* in BMSCs untreated or treated with H_2_O_2_ and/or DEIX (*n* = 4). Letters (a–c) in (**A**,**C**–**F**) indicate significant differences (*p* < 0.05) among the groups in ANOVA followed by Tukey’s multiple comparisons test.

We initially evaluated the effect of DEIX treatment itself on BMSC’ proliferation by CCK-8 assay, in which the DEIX-exposed BMSCs showed a dose-dependent increase in the proliferation until 20 μM of DEIX was added (Figure 2A). We assessed the ability of DEIX to inhibit cellular ROS accumulation in BMSCs exposed to H_2_O_2_ (250 μM), DEIX (0–20 μM), or both by flow cytometric analysis. The flow cytometric histograms indicated a right shift in DCF-specific signal in H_2_O_2_-exposed BMSCs and its recovery by the addition of DEIX (Figure 2B). The H_2_O_2_-mediated increase in DCF-positive BMSCs and its significant suppression by DEIX were supported by determining the mean percentage of DCF-positive BMSCs (Figure 2C). As the Nrf2/HO-1 pathway plays crucial roles in controlling cellular redox homeostasis, we evaluated how DEIX affects the expression of HO-1 and Nrf2 in BMSCs by Western blot analysis. The immunoreactive band specific to HO-1 was increased by the addition of DEIX in a dose-dependent manner, whereas that increase in Nrf2 was found only in the cells treated with 10 μM DEIX (Figure 2D). The DEIX-mediated increase in the induction of HO-1 protein was not affected by the exposure to 250 μM H_2_O_2_ (Figure 2E). To more understand the role of DEIX on the Nrf2/HO-1 pathway, we evaluated the mRNA expression patterns of HO-1 and Nrf2 in the presence and absence of H_2_O_2_ (250 μM) and/or DEIX (0–20 μM) by qRT-PCR (Figure 2F). In parallel with the immunoblot results, the level of *HO-1* in BMSCs was significantly augmented by the addition of DEIX regardless of the presence of H_2_O_2_. The combined treatment with DEIX also significantly restored the expression of *Nrf2* in H_2_O_2_-treated BMSCs, whereas that expression was still lower than that in the control or the cells supplied with DEIX alone. These results indicate that the in vitro antioxidant ability of DEIX was at least in part associated with its activity to directly scavenge cellular ROS, as well as to stimulate the Nrf2/HO-1 pathway.

### 3.3. The Direct Addition of DEIX Restores the H_2_O_2_-Mediated Decrease in BMSC’s Potency to Differentiate into Mineralized Cells

**Figure 3:**
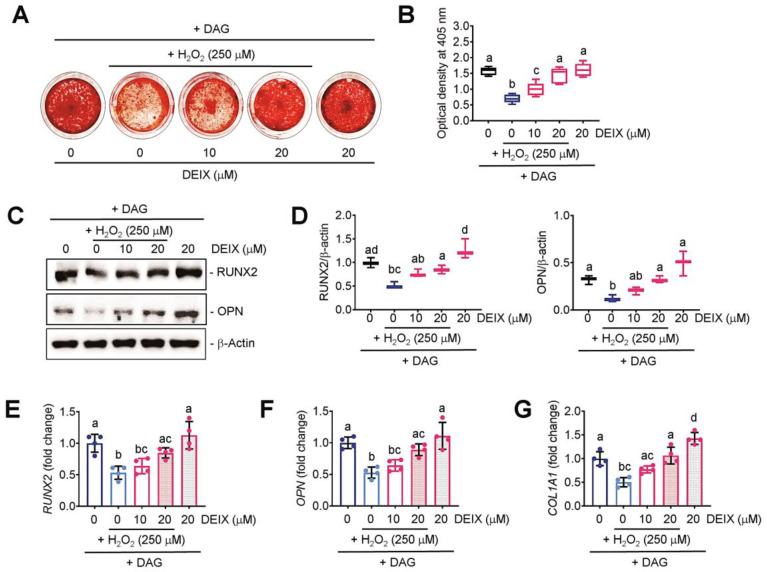
The direct addition of DEIX increases the mineralization of H_2_O_2_-exposed BMSCs by recovering the expression of osteogenic molecules. (**A**) ARS staining images of BMSCs exposed to DAG, H_2_O_2_, and/or DEIX at 21 days post-incubation and (**B**) the optical density corresponding to the dye at 405 nm (*n* = 5). (**C**) Immunoblot data showing the expression patterns of RUNX2 and OPN in BMSCs exposed to H_2_O_2_ and/or DEIX in the presence of DAG for 48 h. (**D**) Densitometric analysis showing the relative expression levels of RUNX2 and OPN to that of β-actin (*n* = 3). The results from qRT-PCR indicating the expression of (**E**) RUNX2, (**F**) OPN, and (**G**) COL1A1 in DAG-treated BMSCs with and without H_2_O_2_ and/or DEIX for 48 h (*n* = 4). Letters (a–d) in panels (**B**,**D**–**G**) indicate significant differences (*p* < 0.05) among the groups in ANOVA followed by Tukey’s multiple comparisons test.

The results from ARS staining exhibited that the exposure to H_2_O_2_ markedly decreased DAG-stimulated mineralization of BMSCs, and that decrease was restored by the addition of DEIX in a dose-dependent manner (Figure 3A). The measurement of optical density specific to the ARS dye supported the H_2_O_2_-mediated reduction in BMSC’s osteogenic ability and its suppression by the direct addition of DEIX (Figure 3B). DEIX treatment alone did not affect the DAG-stimulated mineralization of BMSCs. Western blot data indicated that the H_2_O_2_-mediated decrease in the mineralization and its inhibition by DEIX were accompanied by the DEIX’ potency to recover the expression of osteogenic makers, RUNX2 and OPN (Figure 3C). The densitometric analysis also supported a dose-dependent recovery of these markers via the addition of DEIX (Figure 3D). Similarly, the results from qRT-PCR revealed the downregulation of *RUNX2* (Figure 3E), *OPN* (Figure 3F), and *COL1A1* (Figure 3G) in H_2_O_2_-exposed BMSCs together with a dose-dependent recovery of that downregulation by the addition of DEIX. Specifically, DEIX treatment alone stimulated the expression of *COL1A1*, but not of *RUNX2* and *OPN*, in the BMSCs (Figure 3G). These results highlight that long-term oxidative stress impairs osteogenic potency of BMSCs and that impairment is recovered by the direct addition of DEIX.

### 3.4. The Direct Addition of DEIX Suppresses the H_2_O_2_-Stimulated Osteoclastic Activation of BMMs

**Figure 4:**
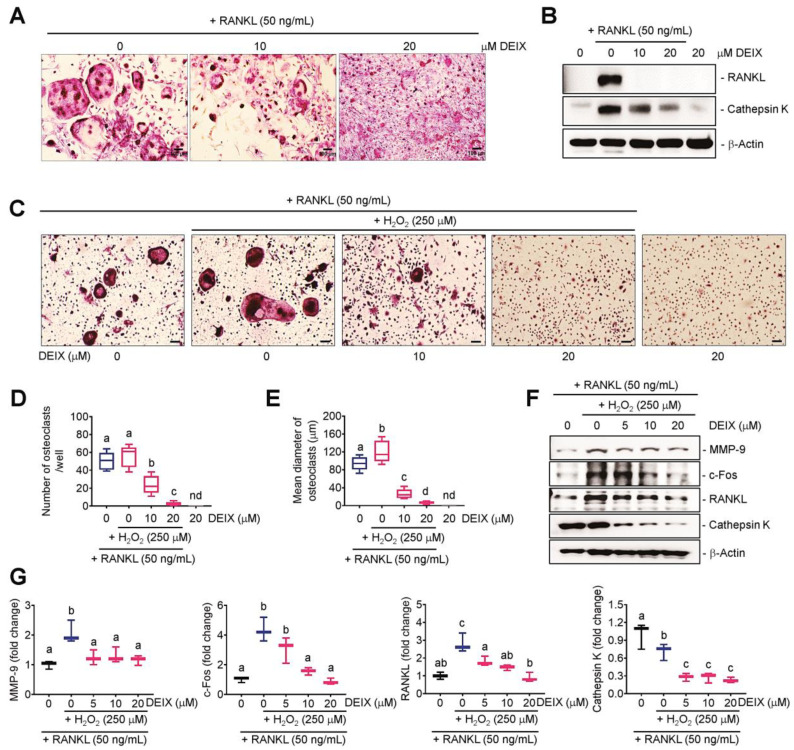
The addition of DEIX suppresses the H_2_O_2_-enhanced osteoclast formation in RANKL-stimulated BMMs by downregulating osteoclastogenic molecules. Young B6 mice-derived BMMs were incubated in the presence and absence of RANKL (50 ng/mL), H_2_O_2_ (250 μM), and DEIX (0–20 μM) and then processed for TRAP staining and immunoblot assays after six and two days of incubation, respectively. (**A**) TRAP staining images showing the RANKL-stimulated osteoclast formation and its suppression by the addition of DEIX. (**B**) Western blot results indicating the expression levels of RANKL and cathepsin K in relation to the presence and absence of RANKL, DEIX, or both. (**C**) Photographs showing TRAP-stained osteoclasts that had formed in BMMs in the presence and absence of RANKL, H_2_O_2_, and DEIX (Scale bar = 50 μm). Analyses of the (**D**) number and (**E**) mean diameter of osteoclasts from the TRAP-stained images (*n* = 5). (**F**) Immunoblot data showing the expression patterns of MMP-9, c-Fos, RANKL, and cathepsin K in BMMs exposed to RANKL, H_2_O_2_, and/or DEIX. (**G**) The graphs showing fold changes in the indicated proteins in relation to the treatment (*n* = 3). Letters (a–d) in panels (**D**,**E**,**G**) indicate significant differences (*p* < 0.05) among the groups in ANOVA followed by Tukey’s multiple comparisons test. nd, not detected.

We determined the effect of DEIX on the H_2_O_2_-enhanced osteoclastic activation and the related mechanisms using BMMs. The direct addition of DEIX suppressed RANKL-stimulated osteoclast formation of BMMs in a dose-dependent manner (Figure 4A). The results from Western blotting indicated that the in vitro anti-osteoclastic potency of DEIX was associated with its ability to downregulate the expression of RANKL and cathepsin K in RANKL-stimulated BMMs (Figure 4B). The addition of DEIX also diminished osteoclast formation by BMMs stimulated with RANKL and H_2_O_2_ (Figure 4C). The numbers and mean diameters of the osteoclasts that formed in combination with RANKL and H_2_O_2_ supported the reagent-mediated osteoclastic stimulation and the dose-dependent anti-osteoclastic potency of DEIX (Figure 4D,E). The immunoblot results indicated that the H_2_O_2_-augmented diameter of the osteoclasts in RANKL-exposed BMMs was associated with the increases in MMP-9, c-Fos, and RANKL, rather than cathepsin K (Figure 4F). In addition, densitometric analysis on the immunoreactive bands corresponding to these osteoclastogenic markers supported the almost complete restoration of the H_2_O_2_-enhanced expression via the addition of DEIX (Figure 4G). Notably, the addition of DEIX extensively diminished the induction of cathepsin K in H_2_O_2_ and RANKL-stimulated BMMs even at the concentration of 5 μM (Figure 4F,G). All these results suggest the potency of DEIX to suppress oxidative stress-enhanced osteoclastic activation in RANKL-stimulated BMMs.

### 3.5. Supplemental DEIX Improves Body and Organ Growths and the Survival Rate of TBI-Exposed Mice

**Figure 5:**
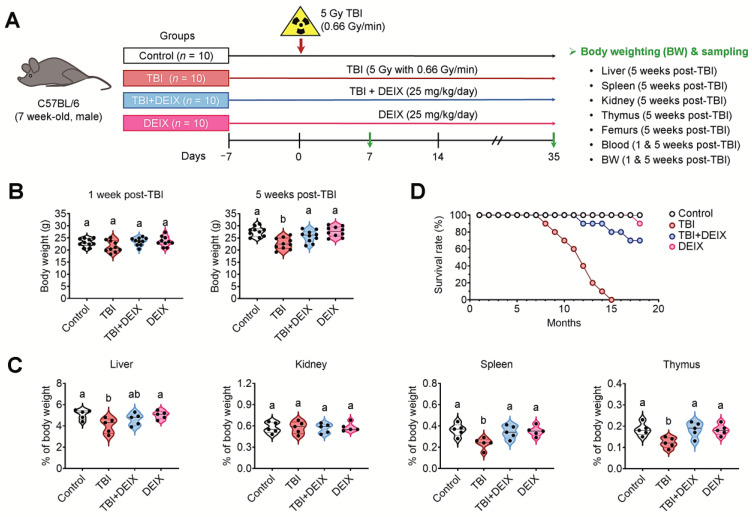
Oral supplementation with DEIX protects mice against TBI-mediated damages to growth and survival. (**A**) Experimental designs for the mouse groups and sample collection 7 and 35 days after TBI. (**B**) Body weights (g) in the mouse groups one and five weeks post-TBI (*n* = 10/group). (**C**) Weights of the indicated organs were measured five weeks post-TBI and are represented as the % of body weight (*n* = 5). (**D**) Survival rates of the mouse groups at the indicated months post-TBI (*n* = 10/group). Letters (a and b) in panels (**B**,**C**) indicate significant differences (*p* < 0.05) among the groups in ANOVA followed by Tukey’s multiple comparisons test.

The experimental designs for the mouse groups, TBI exposure, and DEIX administration along with the collection of samples one and/or five weeks after TBI are shown in Figure 5A. No differences in body weights were found among the groups one week post-TBI, but the TBI group showed significantly lower body weight five weeks post-TBI than either the control, TBI + DEIX, or DEIX group (Figure 5B). When organ weights were compared among the groups, the TBI group had significantly lower weights of the liver, spleen, and thymus compared with the control or DEIX group, but the weight of organs did not differ between the control and TBI + DEIX group (Figure 5C). The TBI + DEIX group had an 80% survival rate until 16 months, whereas all the mice in the TBI group had died by 15 months after TBI (Figure 5D). These results support that TBI defects body and organ growths and shortens lifespan in a mouse model, whereas those defects are recovered by supplementation with DEIX.

### 3.6. Oral Administration of DEIX Inhibits Structural Damage to the Organs of TBI-Exposed Mice

**Figure 6:**
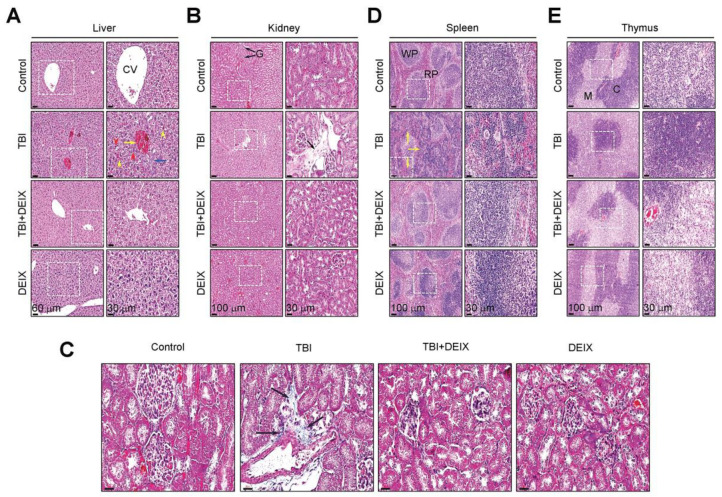
Supplemental DEIX inhibits structural alterations of organs in a mouse model of TBI exposure. IHC staining images of (**A**) liver, (**B**) kidney, (**D**) spleen, and (**E**) thymus sections. CV, central vein; G, glomerulus; WP, white pulp; RP, red pulp; M, medulla; C, cortex. Yellow arrow, blue arrow, red arrow heads, and yellow arrow heads in panel (**A**) indicate central vein congestion, fibrotic cells, blood sinusoids, and apoptotic/necrotic cells, respectively. Black arrows in panel (**B**) exhibit glomerulus and a structural change around the glomerulus. Yellow arrows in panel (**D**) indicate TBI-mediated structural destruction of white and red pulp regions. Panel (**C**) shows the MT staining images exhibiting a collagenous change in kidney of TBI-exposed mice (Scale bar = 30 μM). Black arrows indicate the fibrotic tissues formed in kidney of TBI-exposed mice.

Dissimilar to the control group, the TBI group expressed morphological characteristics such as central vein congestion (yellow arrow), blood sinusoids (yellow arrow heads), fibrotic cells (blue arrow), and apoptotic/necrotic cells (red arrow heads) in their liver tissue five weeks post-TBI (Figure 6A). However, the TBI-derived morphological characteristics were not found in the liver of the TBI + DEIX group. TBI exposure also caused the formation of collagenous fibrotic tissue in the kidney without a morphological change in the glomerulus, but that formation disappeared almost completely when the mice received DEIX supplementation (Figure 6B). TBI-mediated fibrosis in the kidney and its suppression by supplemental DEIX was supported by MT staining (Figure 6C). In addition, TBI exposure led to the structural destruction of white and red pulp regions in the spleen, but that destruction was not found in spleens from the control, TBI + DEIX, and DEIX groups (Figure 6D). However, all the mouse groups exhibited normal structures in their thymic medulla and cortex regions (Figure 6E). These results indicate that the liver, kidney, and spleen had greater radiosensitivity than the thymus, and the TBI-derived tissue damages are recovered by the long-term administration of DEIX.

### 3.7. The Recovery of TBI-Mediated Liver Damage by DEIX Was Accompanied by the Restoration of Antioxidant Defense Systems and Decreased TNF-α Levels

**Figure 7:**
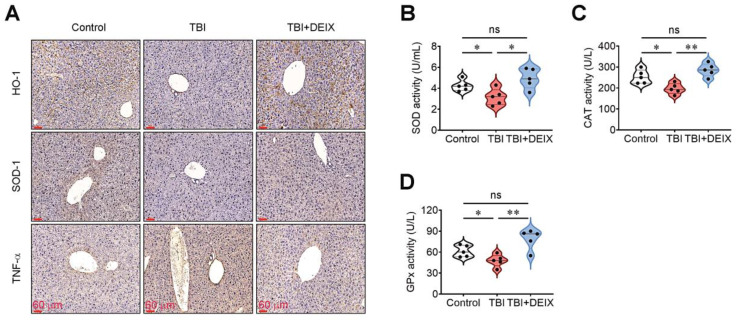
Supplemental DEIX restores the antioxidant defense system and diminishes the induction of inflammatory cytokine in the livers of TBI-exposed mice. (**A**) IHC staining images exhibiting the expression patterns of HO-1, SOD-1, and TNF-α in liver tissues from the control, TBI, and TBI + DEIX groups at five weeks post-TBI. The enzymatic activities of (**B**) SOD, (**C**) CAT, and (**D**) GPx in liver homogenates from the mouse groups at five weeks post-TBI (*n* = 5). * *p* < 0.05 and ** *p* < 0.01 in an unpaired Student’s *t*-test. ns, not significant.

We next analyzed the expression patterns of antioxidation- and inflammation-associated molecules in the liver of the mouse groups via IHC staining. Compared with the control group, the TBI group expressed transparently lower levels of HO-1 and SOD-1 and a greater level of TNF-α in liver tissue, whereas these changes were restored by supplemental DEIX up to levels similar to those in the control group (Figure 7A). In parallel with that finding, the TBI group-derived liver tissue contained significantly lower SOD (Figure 7B), CAT (Figure 7C), and GPx activities (Figure 7D) than that from the control group. The TBI-mediated decreases in these enzyme activities were completely recovered by the oral administration of DEIX (Figure 7A–C). These results support that the liver tissue is very sensitive to damage during radiotherapy [18,31] and indicate that this damage is orchestrated by the decreased antioxidant activity and increased inflammatory response in the liver. These findings also postulate that the DEIX-mediated recovery of TBI-derived liver damages may contribute to the improved survival rate in TBI-exposed mice.

### 3.8. Supplemental DEIX Restores the TBI-Induced Impairment in Hematopoietic Development and BM Microenvironment

**Figure 8:**
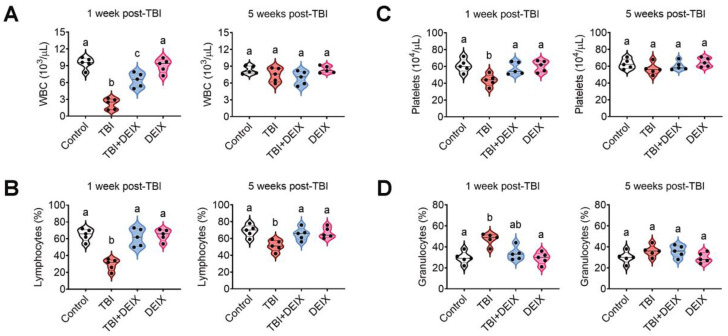
Supplemental DEIX recovers the abnormal production of circulating blood cells in TBI-exposed mice. Determination of circulating (**A**) WBCs, (**B**) lymphocytes, (**C**) platelets, and (**D**) granulocytes in the mouse groups using an automated complete blood cell counter at one and five weeks post-TBI (*n* = 5). Letters indicate significant differences (*p* < 0.05) among the groups in ANOVA followed by Tukey’s multiple comparisons test.

**Figure 9:**
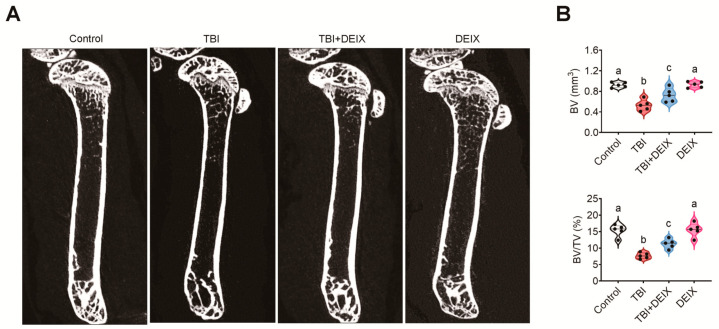
Supplemental DEIX recovers BM microenvironmental impairment in TBI-exposed mice. (**A**) The 2D μCT images of the femurs derived from the indicated mouse groups. (**B**) The values of BV (mm^3^) and BV/TV (%) (*n* = 5). Letters in panel (**B**) indicate significant differences (*p* < 0.05) among the groups in ANOVA followed by Tukey’s multiple comparisons test.

Because TBI time-dependently causes the functional loss of BM-residing HSCs by inducing oxidative stress [32,33], we determined the effect of supplemental DEIX on the hematopoietic process by analyzing the compositions of circulating blood cells one and five weeks after TBI. The TBI group exhibited significantly fewer WBCs (Figure 8A), lymphocytes (Figure 8B), and platelets (Figure 8C) and more granulocytes (Figure 8D) than the control, TBI + DEIX, or DEIX group one week post-TBI. A relatively lower level of lymphocytes, without a change in the number of other circulating blood cells, was also found in the TBI group, compared with the other mouse groups, at five weeks post-TBI (Figure 8A–D, right panels). We also explored whether supplemental DEIX protects the TBI-mediated impairment in BM microenvironment. Figure 9A shows the results from 2D μCT analysis and indicates lower bone mass in the TBI group than in the control, TBI + DEIX, or DEIX group five weeks post-TBI. When the bone parameter values were evaluated, the TBI group had significantly lower BV and BV/TV values than the control group, and those decreases were markedly restored in the TBI + DEIX group (Figure 9B). These results support that TBI exposure acutely damages hematopoietic development, and that damage can be prevented or protected by the supplementation with DEIX. The current findings also suggest that TBI-mediated impairment in BM microenvironment is recovered by the long-term supplementation with DEIX.

## 4. Discussion

As evidenced by the results from CCK-8 and flow cytometry, our results demonstrate that the addition of DEIX directly stimulates the proliferation and inhibits ROS accumulation in H_2_O_2_-exposed BMSCs. Our findings also highlight that the H_2_O_2_-stimulated BMSCs exhibit the decrease in Nrf2, but the increase in HO-1 expression, whereas that changes are differently affected by the addition of DEIX. In general, the Nrf2/HO-1 pathway plays key roles in the cellular response to oxidative stress, but they may have distinct roles [34]. Nrf2 is translocated to the nucleus and leads to the expression of various antioxidant and cytoprotective genes, including HO-1, whereas an enzymatic HO-1 degrades heme and produces the byproducts that act as antioxidant and anti-inflammatory molecules [35,36]. ROS also act as signaling molecules to activate the Nrf2 pathway, by which the expression of HO-1 is upregulated [37,38]. However, it is important to consider that in addition to ROS, the Nrf2 and HO-1 respond to other stressors, in which the activity of HO-1 directly affects cellular redox balance by metabolizing heme, while Nrf2 controls a broader range of cellular processes [39,40]. Accordingly, we consider that DEIX has the potency to directly remove cellular ROS without a cytotoxic effect, and that potency is partially associated with the activation of Nrf2/HO-1 pathway. Further detail experiments to clarify the exact roles of the Nrf2/HO-1 pathway in DEIX- and/or H_2_O_2_-exposed BMSCs are required.

An imbalance between osteoclast and osteoblast activity leads to excessive bone resorption under a condition of oxidative stress or inflammation [41]. Osteoblast activity is tightly affected by osteogenesis-specific transcription factors including RUNX2, osterix, and bone morphogenetic protein 2 together with their downstream effectors, such as COL1A1, OPN, and OCN [42]. RUNX2 plays a crucial role in maintaining bone homeostasis, and thus the disruptions in RUNX2 expression or function enhance bone resorption along with the structural and functional destruction of bones [43]. RANKL is a key signaling molecule that triggers the differentiation and maturation of osteoclasts via the interaction with its receptor, RANK [44]. Cathepsin K is a key enzyme in osteoclasts and produced in proportion to osteoclast activity, and thus its level suggests the progression of physiological bone resorption [45,46]. c-Fos and MMP-9 also play crucial roles in osteoclast formation and function. For example, a component of activator protein-1, c-Fos, is induced by RANKL and directly regulates the expression of several genes critical for osteoclast development [47]. MMP-9 is expressed in osteoclasts and plays a complex role in osteoclast formation and bone remodeling, as well as in the degradation of bone matrix components [48]. Accordingly, our current findings support the potency of DEIX to recover the H_2_O_2_-induced imbalance between osteoblastic and osteoclastic activation of BM-derived cells. Our results also provide the associated mechanism by which the addition of DEIX recovers the imbalanced osteoblastic and osteoclastic activities in oxidative stress-exposed stem-like cells.

In this study, we examined the radioprotective effects of supplemental DEIX using a mouse model of TBI exposure. To this end, we applied 5 Gy as the single TBI sublethal dose. This is because that the dose at the range from 4 to 8 Gy reduces the number of BM cells within a few days and the total number of Lin^−^Sca-1^+^cKit^hi^ cells representing lymphocyte progenitors in the BM remain reduced 2 months post-TBI [49,50]. However, TBI dose of more than 9 Gy can cause mice to die 14 days after TBI, if a reconstitution is absent [50].

TBI can cause severe damage to intact soft and hard tissues, depending on the doses and times applied [51,52]. TBI-mediated damage includes the decrease in body growth, the excessive accumulation of intracellular ROS, the functional loss of BM stem cells, and abnormal hematopoietic development [14,16,49,53]. Thus, TBI-induced disorders are similar to the hallmarks of age-related degenerative complications [54]. The TBI-mediated BM damage immediately generates ROS in HSCs, and that generation induces HSC senescence and long-term BM injury [14,17]. The TBI-derived defects in HSCs and BM microenvironment cause abnormal hematopoietic development, in which the decreased levels of WBCs and lymphocytes and/or their delayed recovery may contribute to the reduced survival rate [49]. TBI impairs the structures or functions of liver, spleen, and kidney [30,31,55], and that impairment also increases the lethality of TBI-exposed animals. However, further investigation to elucidate the long-term pathophysiological consequences of TBI and the effects of DEIX in relation to the TBI-derived mortality will be necessary.

We previously found that phenolic acids such as caffeic acid, ferulic acid, and coumaric acid protect against TBI-mediated mortality in an animal model by recovering TBI-induced oxidative injuries [23,49,56]. It is also found that astaxanthin, a kind of carotenoid, ameliorates TBI-induced oxidative damage to the hematopoietic system [57]. In parallel with previous reports, we found that the exposure to TBI induces age-associated complications in organs along with the impairments in growth, survival, and hematopoietic progression. We also indicate the association of TBI-induced liver damages with the impaired antioxidant defense system and the increased inflammatory response. In addition, our current findings not only support a close association of BM microenvironmental impairment with the abnormal hematopoietic development in TBI-exposed mice, but also indicate the recovery of that impairment via the supplementation with DEIX. Taken as a whole, our findings suggest the beneficial effects of long-term DEIX supplementation on TBI-mediated local and systemic complications. Furthermore, our results highlight that the radioprotective potency of bioactive antioxidants is closely associated with their capacities to recover or prevent the induction of senescence and oxidative stress in BM-residing stem cells, abnormal hematopoietic development, BM impairment, and functional disorders of TBI-sensitive organs. Further detail experiments to elucidate the effects of supplemental DEIX on the BM and BM-residing stem cells in TBI-exposed mice will be necessary. Moreover, future studies should incorporate proper experimental design and statistical methodology to ensure robust and reproducible results in the survival study.

## 5. Conclusions

In summary, this study introduces the methods to efficiently produce DEIX and highlights its biological potential to suppress H_2_O_2_-stimulated oxidative stress without a cytotoxic effect. Our results show the ability of DEIX to recover the oxidative stress-stimulated imbalance between osteoblast and osteoclast activities in BM-derived mesenchymal or hematopoietic lineage cells. The current findings also highlight that a long-term supplementation with DEIX protects mice against TBI-mediated impairments in growth, organ, survival, hematopoietic progression, and BM microenvironment. Overall, this study supports the biological efficacy of DEIX to recover oxidative cellular complication and functional loss of BM-derived cells along with its efficacy as a pharmaco-therapeutic antioxidant.

## Data Availability

The data that support the findings of this study are available from the corresponding author upon reasonable request.
