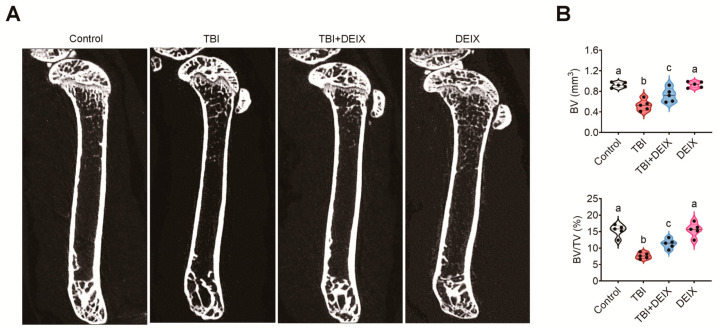# Correction: Bhattarai et al. Deinoxanthin Recovers H_2_O_2_-Stimulated Oxidative Complications of Bone Marrow-Derived Cells and Protects Mice from Irradiation-Mediated Impairments. *Antioxidants* 2025, *14*, 1180

**DOI:** 10.3390/antiox15040514

**Published:** 2026-04-21

**Authors:** Govinda Bhattarai, Sung-Ho Kook, Saroj Kumar Shrestha, Jeong-Hwan Park, Shankar Rijal, Gyeongho Tae, Doyoung Hwang, Seung-Moon Park, Jeong-Chae Lee, Young-Mi Jeon

**Affiliations:** 1 Cluster for Craniofacial Development and Regeneration Research, Institute of Oral Bioscience, School of Dentistry, Jeonbuk National University, Jeonju 54896, Republic of Korea; govinda@jbnu.ac.kr (G.B.); kooksh@jbnu.ac.kr (S.-H.K.); choosey95@naver.com (J.-H.P.); 2 Research Center of Bioactive Materials, Department of Bioactive Material Sciences, Jeonbuk National University, Jeonju 54896, Republic of Korea; rijal49@jbnu.ac.kr; 3 Department of Biochemistry and Molecular Genetics, University of Alabama at Birmingham, Birmingham, AL 35233, USA; skshrest@uab.edu; 4 Department of Bioenvironmental Chemistry, Jeonbuk National University, Jeonju 54896, Republic of Korea; rudgh2360@jbnu.ac.kr (G.T.); hdy0928@jbnu.ac.kr (D.H.); smpark@jbnu.ac.kr (S.-M.P.); 5 Research Institute of Clinical Medicine of Jeonbuk National University, Biomedical Research Institute of Jeonbuk National University Hospital, Jeonju 54907, Republic of Korea

In the original publication [1], there was a mistake in Figure 9A (2D μCT images of the femurs derived from the indicated mouse groups). Figure 9A contains a mistake derived from the incorrect use of a 2D μCT image for the DEIX group. The corrected Figure 9A appears below. The authors state that the scientific conclusions are unaffected. This correction was approved by the Academic Editor. The original publication has also been updated.